# Current Status and Future Prospects of Research on Sepsis-Related Acute Kidney Injury

**DOI:** 10.3390/ijms27031315

**Published:** 2026-01-28

**Authors:** Yurou Wang, Le Zong, Manli Zhu, Jie Li, Jiayi Xu, Hunian Li, Yan Li

**Affiliations:** 1 Department of Emergency Medicine, Tongji Hospital, Tongji Medical College, Huazhong University of Science and Technology, Wuhan 430030, Chinazledoctor@163.com (L.Z.);; 2 Department of Critical Care Medicine, Tongji Hospital, Tongji Medical College, Huazhong University of Science and Technology, Wuhan 430030, China

**Keywords:** sepsis, AKI, signaling pathways, targeted therapies

## Abstract

Sepsis is defined as life-threatening organ dysfunction caused by a dysregulated host response to infection. The kidney is among the organs most susceptible to sepsis-induced injury, and acute kidney injury frequently develops in this context, thereby markedly increasing mortality in affected patients. With continued advances in research, a more comprehensive understanding has been achieved regarding the clinical risk factors, pathophysiological mechanisms, therapeutic responses, and renal recovery processes associated with sepsis-associated acute kidney injury (SA-AKI). These advances have strengthened the capacity for prevention, early detection, and effective management of SA-AKI. Despite this progress, substantial gaps remain in the overall understanding of SA-AKI pathogenesis, including the complex interplay among pathophysiological mechanisms and the extensive cross-regulation of multiple signaling pathways. Consequently, SA-AKI remains a major clinical challenge and imposes a substantial global healthcare burden. There is therefore an urgent need for further research to elucidate the underlying mechanisms of SA-AKI and to identify more effective therapeutic strategies. Unlike previous reviews that primarily focused on individual mechanisms or isolated therapeutic targets, the present review synthesizes the most recent evidence on SA-AKI. Particular emphasis is placed on its pathogenic processes, associated molecular mechanisms and signaling pathways, and emerging therapeutic targets. Special attention is given to the hierarchical relationships among distinct mechanisms during disease progression and their implications for clinical translation. This review aims to inform clinical practice and to identify future research directions, thereby providing valuable insights for both researchers and clinicians in this field.

## 1. Introduction

Sepsis is a leading cause of critical illness in intensive care units (ICUs), with its incidence and mortality rates remaining high and continuing to increase, thereby representing a substantial global threat to human health [1]. The Third International Consensus Definition for Sepsis and Septic Shock (Sepsis-3), released in 2016, defines sepsis as life-threatening organ dysfunction caused by a dysregulated host response to infection [2]. Epidemiological data suggest that up to 60% of patients with sepsis develop acute kidney injury (AKI), making sepsis one of the most common etiologies of AKI in the ICU setting [3,4]. According to the Kidney Disease: Improving Global Outcomes (KDIGO) guidelines and the Third International Consensus Definition for Sepsis and Septic Shock (Sepsis-3), sepsis-associated acute kidney injury (SA-AKI) is defined as acute kidney injury occurring within 7 days of sepsis diagnosis. SA-AKI is further classified into early-onset SA-AKI, defined as AKI developing within 48 h of sepsis diagnosis, and late-onset SA-AKI, defined as AKI occurring between 48 h and 7 days after sepsis diagnosis [5]. The diagnosis of sepsis is established according to the Sepsis-3 criteria, with the Sequential Organ Failure Assessment (SOFA) score used as an adjunctive tool. The diagnosis of acute kidney injury (AKI) is based on the Kidney Disease: Improving Global Outcomes (KDIGO) criteria, which include an increase in serum creatinine of more than 0.3 mg/dL (26.5 μmol/L) within 48 h or an increase to at least 1.5 times the baseline value within 7 days, as well as a urine output of less than 0.5 mL/kg/h persisting for more than 6 h [5]. However, SA-AKI is not equivalent to isolated ischemic or nephrotoxic AKI. Elevations in serum creatinine may be delayed, and although changes in urine output are sensitive indicators, they lack specificity. Non-oliguric AKI is common in patients with SA-AKI, whereas persistent oliguria is associated with a poor prognosis.

Owing to historically limited diagnostic criteria, heterogeneity in clinical patient populations, and variability in clinical practice settings worldwide, the epidemiological characteristics of SA-AKI exhibit substantial variation. Reported incidence rates of SA-AKI range from 14% to 87%, while associated mortality rates—including ICU mortality, in-hospital mortality, 28-day mortality, and 90-day mortality—range from 11% to 77% [5]. These variations largely reflect differences in AKI diagnostic criteria, the evolution of sepsis definitions, and heterogeneity in study populations and clinical settings. Early studies often relied on less sensitive diagnostic definitions or exclusively on serum creatinine measurements, leading to frequent underestimation of AKI incidence. In contrast, contemporary ICU-based studies employing Kidney Disease: Improving Global Outcomes (KDIGO) criteria and including urine output monitoring typically report substantially higher incidence rates. Moreover, the proportion of ICU patients diagnosed with SA-AKI has demonstrated a steady annual increase. Compared with the general ICU population, patients with SA-AKI tend to present with more severe and complex clinical conditions, require more extensive multi-organ support, and pose greater therapeutic challenges [6,7]. Current treatment strategies for SA-AKI remain primarily limited to traditional anti-inflammatory and supportive therapies. As a result, the widespread use of antibiotics may contribute to AKI development through mechanisms such as direct tubular toxicity, oxidative stress, and immune-mediated interstitial nephritis, with variable degrees of reversibility.

In recent years, substantial progress has been achieved in molecular-level investigations into the pathogenesis and pathophysiology of SA-AKI. Accumulating evidence indicates that SA-AKI is not solely attributable to acute tubular necrosis, but instead represents a complex and dynamic process involving multiple interacting factors, including microcirculatory disturbances, exaggerated inflammatory responses, and metabolic remodeling of renal tubular epithelial cells. Nevertheless, significant knowledge gaps remain, including the mechanisms by which microcirculatory dysfunction and inflammation influence one another, the existence of network interactions among distinct pathophysiological processes, the cross-regulation of multiple signaling pathways, and the translation of mechanistic findings into effective clinical interventions. Accordingly, a comprehensive understanding of the signaling pathways involved in SA-AKI development is essential for elucidating its complex pathophysiological mechanisms. This review integrates current evidence on the pathophysiology and signaling mechanisms underlying SA-AKI, highlighting emerging research priorities in the field. These include key molecules, signaling pathways, and biomarkers that may represent potential targets for novel therapeutic development. Both established and emerging signaling pathways reported in the literature are categorized by function, encompassing four major domains: hemodynamic alterations, excessive inflammatory responses, energy metabolism reprogramming, and mechanisms of cell death. By detailing the localization and mechanisms of action of these pathways at the tissue, cellular, and organelle levels, their roles in the pathophysiological progression of SA-AKI are highlighted. Furthermore, recent advances in potential therapeutic strategies targeting specific signaling pathways are reviewed. These therapeutic approaches aim to modulate pathophysiological processes across the aforementioned four domains and to improve efficacy in preclinical models of SA-AKI by targeting specific molecules or regulating relevant signaling pathways. Collectively, these findings not only deepen the understanding of SA-AKI but also offer valuable guidance for the development of more effective therapeutic strategies in the future.

In summary, this review aims to provide a systematic synthesis of the key pathophysiological mechanisms underlying SA-AKI for translational medicine researchers and clinical scientists involved in sepsis and acute kidney injury research, with particular emphasis on their relevance to clinical diagnosis and therapeutic translation. The molecular and cellular mechanisms discussed herein are not presented in isolation but are intended to illuminate practical challenges, including clinical heterogeneity, diagnostic complexity, and limitations in therapeutic translation. Collectively, these insights are expected to advance both basic research and clinical practice in SA-AKI, ultimately contributing to reductions in disease incidence and mortality and to improvements in patient quality of life.

## 2. Pathophysiology

The pathophysiological mechanisms of SA-AKI encompass multiple interrelated processes, including hemodynamic alterations, excessive inflammatory responses, energy metabolism reprogramming, and mechanisms of cell death. These processes do not occur in isolation but are highly interconnected and act in a synergistic manner, thereby collectively driving the progression of renal injury.

### 2.1. Hemodynamics

#### 2.1.1. Macrocirculation

Hemodynamic disturbances in SA-AKI manifest as combined impairments of both large vessels and the microcirculation. Patients with sepsis frequently exhibit characteristic hemodynamic dysregulation: systemic vasodilation coupled with a reduction in effective circulating volume results in marked reductions in renal blood flow and inadequate perfusion, subsequently causing renal dysfunction [8]. Importantly, recent studies indicate that, despite increased cardiac output and renal blood flow in some patients with hyperdynamic septic shock, regional renal hypoperfusion may persist. This heterogeneity in renal perfusion is particularly evident histologically, with the medullary region demonstrating marked vulnerability to hypoperfusion [9]. The resulting regional imbalance in blood flow distribution can disrupt the tubular microenvironment, thereby leading to structural damage. At the hemodynamic level, the characteristic decline in glomerular filtration rate (GFR) observed in SA-AKI patients is clinically significant. Hemodynamic studies reveal that this alteration is strongly associated with differences in afferent and efferent arteriolar dilation: when efferent arteriolar dilation exceeds that of afferent arterioles, the effective glomerular filtration pressure decreases substantially, resulting in a pathological “high perfusion–low filtration” state [10].

#### 2.1.2. Microcirculation

Evidence indicates that even in the absence of observable macrovascular hemodynamic changes, microcirculatory alterations can arise during sepsis and play a pivotal role in the progression of sepsis-induced organ injury [11]. These microcirculatory abnormalities primarily manifest as pronounced heterogeneity in microvascular flow [12]. This disorder impairs renal function through two principal mechanisms: first, it induces an imbalance between tissue oxygen supply and demand, leading to bioenergetic and metabolic disturbances [13]; second, it promotes a cascade of inflammatory mediators and vasoactive substances. Studies have demonstrated significantly elevated levels of molecular markers, including soluble adhesion molecules, angiopoietin-2, and its receptors, in the serum of septic patients [14]. These alterations synergize with inflammatory cytokines to disrupt microvascular endothelial function, resulting in key pathological features such as enhanced leukocyte–platelet rolling adhesion and impaired molecular transport [11]. Microvascular endothelial dysfunction further exacerbates renal injury through multiple mechanisms: first, it impairs blood flow autoregulation, causing perfusion imbalance; second, it disrupts the hemostatic–anticoagulant equilibrium, promoting microthrombus formation; third, it increases vascular permeability, inducing tissue edema; and fourth, it amplifies the inflammatory response through abnormal leukocyte recruitment [15]. This multi-targeted functional impairment not only exacerbates local hypoxia and energy metabolism disorders but also establishes a vicious cycle of inflammation-induced ischemia, thereby promoting the progression of SA-AKI toward an irreversible state.

Beyond abnormal blood perfusion, renal tubular epithelial cells act as active sensors and signal amplifiers of microcirculatory disturbances. Studies indicate that tubular injury or mechanical stress triggers the release of extracellular nucleotides, including ATP and UTP. These signals activate receptors to regulate cytoskeletal remodeling and oriented cell migration. Furthermore, research by Gessler et al. demonstrates that nucleotide signaling not only mediates post-injury migratory responses of renal tubular epithelial cells but may also modulate local interactions between the vasculature and epithelium, thereby translating microcirculatory disturbances into cellular responses [16].

Consequently, relative to traditional models, the renal perfusion heterogeneity resulting from large-vessel hemodynamic disturbances may contribute to the limited efficacy of conventional renal replacement therapies in SA-AKI. Furthermore, the interplay between microcirculatory dysfunction and inflammatory responses constitutes a particularly complex component of the pathophysiological mechanisms underlying SA-AKI.

### 2.2. Inflammations

Inflammatory responses represent central drivers of SA-AKI [17,18]. Activation of Toll-like receptors (TLRs), such as TLR-2 and TLR-4, and the NF-κB pathway by pathogen-associated molecular patterns (PAMPs) or damage-associated molecular patterns (DAMPs) induces a robust proinflammatory cytokine response (e.g., IL-1β, IL-6, IL-8, and TNF-α). The dual expression of TLR-2 and TLR-4 in the kidney is particularly important, as these receptors are expressed not only in immune cells, including monocytes and macrophages, but also on the surface of renal tubular epithelial cells [11,19]. Receptor activation triggers NF-κB nuclear translocation via MyD88-dependent signaling, resulting in the rapid release of proinflammatory factors. This excessive inflammatory response mediates renal parenchymal injury through two principal pathways: first, by directly inducing pyroptosis and necrosis of tubular epithelial cells; and second, by activating peritubular capillary endothelial cells, which leads to upregulation of adhesion molecules (ICAM-1 and VCAM-1) and transendothelial migration of inflammatory cells (neutrophils and monocytes), resulting in interstitial inflammatory infiltration [20]. Importantly, the microcirculatory dysfunction characteristic of SA-AKI exhibits a mutually reinforcing interaction with the inflammatory response: abnormal blood flow distribution promotes leukocyte activation through altered shear stress, while the local inflammatory microenvironment exacerbates endothelial dysfunction. Together, these factors activate oxidative stress and mitochondrial-dependent apoptosis pathways, thereby establishing a self-perpetuating pathological cycle of inflammation, ischemia, and recurrent injury. Beyond passive injury, renal tubular epithelial cells actively sense and respond to microenvironmental stress through nucleotide-dependent signaling mechanisms. Following tubular injury, hypoxia and microcirculatory dysfunction stimulate the release of extracellular nucleotides, such as ATP. These nucleotides function as signaling molecules via purinergic receptors, regulating the oriented migration of tubular epithelial cells and coordinating local inflammatory responses. Recent research by Gessler et al. demonstrates that nucleotide signaling is critical for directing post-injury epithelial cell behavior, thus linking microcirculatory disruption to inflammatory activation. This mechanism underscores the dual, context-dependent role of tubular epithelial cells in propagating injury and facilitating repair during SA-AKI [16].

Despite extensive experimental evidence demonstrating the pivotal role of inflammation in SA-AKI, the efficacy of clinical anti-inflammatory therapies remains limited. This suggests that inflammation primarily acts as an amplifying mechanism rather than as the sole initiating factor, with its pathogenic effects modulated by microcirculatory status, metabolic context, and the extent of activation in cell death pathways. Consequently, therapeutic strategies targeting a single inflammatory pathway may be insufficient to achieve optimal clinical outcomes.

### 2.3. Energy Metabolism Reprogramming

Sepsis disrupts cellular metabolic pathways, resulting in the reprogramming of cellular energy metabolism. These metabolic alterations may either enhance cellular adaptation and repair processes or precipitate cell death. Mitochondria, as the central organelle for energy metabolism, have been shown to undergo dysfunction in the sheep SA-AKI model by Tomas Luther and colleagues [21]. Serum and urine metabolomics studies based on nuclear magnetic resonance technology further reveal that key mitochondrial pathways involved in tubular energy metabolism are disrupted in sepsis. These metabolic alterations are strongly associated with changes in renal function [22]. Severe mitochondrial damage exacerbates oxidative stress and energy metabolism disturbances. Notably, reactive oxygen species (ROS) production significantly increases, accompanied by concurrent changes in renal nitric oxide levels and nitrosative stress, thereby perturbing the redox balance [23]. This stress further damages renal tubular epithelial cells, resulting in functional impairment and ultimately cell death. N-acetylcysteine (NAC), an effective ROS scavenger, has been shown to confer nephroprotective effects, confirming the central role of oxidative stress. This drug restores redox balance by replenishing glutathione pools, thus interrupting the self-perpetuating cycle of microcirculatory impairment and tubular injury [24,25].

In SA-AKI, tubular epithelial cells exhibit altered utilization of metabolic substrates, including glucose, amino acids, fatty acids, ketone bodies, citrate, and lactate [26]. This metabolic reprogramming manifests not only as downregulation of fatty acid oxidation and upregulation of glycolysis but also encompasses alterations in membrane lipids, triglycerides, the pentose phosphate pathway, lipogenesis, and amino acid metabolism. These adjustments in energy metabolism pathways, concomitant changes in metabolite levels, and shifts in key enzyme activities may further modulate cell death pathways in tubular epithelial cells, including autophagy, apoptosis, necroptosis, and ferroptosis [27]. This complex metabolic remodeling not only reflects the kidney’s adaptive response to sepsis-induced stress but also underscores its potential as a therapeutic target. Modulating these metabolic changes may offer novel strategies for mitigating SA-AKI.

### 2.4. Cell Death

Cell death can be broadly categorized into two primary categories: programmed cell death and non-programmed cell death. Non-programmed cell death constitutes an unregulated biological process, typically exemplified by necrosis. This type of cell death is often directly triggered by external physical or chemical injury, without intrinsic regulatory control. It results in rapid disintegration of cellular structures and release of cellular contents, potentially inducing inflammatory responses in surrounding tissues. Therefore, this form of cell death is beyond the scope of this review. Programmed cell death encompasses caspase-dependent apoptosis, non-caspase-dependent necroptosis, pyroptosis, autophagy, and ferroptosis [28]. However, simplistically categorizing cell death as “programmed” or “non-programmed” has limitations in the current research context. Recent studies indicate that multiple forms of cell death (e.g., necroptosis, pyroptosis, and ferroptosis), though regulated by specific molecular pathways and considered “regulated” cell death, exhibit morphological and inflammatory characteristics similar to classical necrosis. In sepsis, these multiple cell death pathways are concomitantly activated, collectively contributing to renal dysfunction [20,29,30,31].

#### 2.4.1. Apoptosis

Lerolle et al. performed renal biopsies on 19 patients who succumbed to septic shock accompanied by anuric acute kidney injury. They observed markedly elevated tubular cell apoptosis and capillary leukocyte infiltration in these patients, providing strong evidence for apoptosis as a key pathological mechanism in SA-AKI [32]. Its pathogenesis involves multifactorial interactions: systemic circulatory dysfunction-induced ischemia-hypoxia, excessive inflammatory response, and metabolic stress collectively constitute the pathological basis for apoptosis induction [33]. Notably, metabolically associated epigenetic regulation—specifically histone lactylation—is markedly elevated in SA-AKI. Jiao Qiao and colleagues demonstrated that histone lactylation enhances inflammation and apoptosis in SA-AKI, thereby exacerbating renal dysfunction [34].

Necrotic apoptosis represents another form of programmed cell death. Its morphological features resemble necrosis, including cell swelling, membrane rupture, and release of cellular contents, frequently accompanied by inflammatory responses [35]. Unlike caspase-mediated apoptosis, necrotic apoptosis does not depend on caspase activation. Instead, it mediates cell death through interactions between RIPK1 and RIPK3, along with phosphorylation and membrane translocation of MLKL [36]. In vivo and in vitro experiments on SA-AKI demonstrated markedly increased expression of p-RIPK3, RIPK3, and MLKL in LPS-stimulated HK-2 cells and kidney tissues from septic mice. These findings suggest that necrotic apoptosis in renal tubular epithelial cells contributes to the pathogenesis and progression of SA-AKI [37,38].

#### 2.4.2. Pyroptosis

Pyroptosis is a pro-inflammatory form of programmed cell death that is mediated via two distinct pathways in SA-AKI: the canonical pathway, dependent on Caspase-1, and the non-canonical pathway, involving direct activation of Caspase-4, -5, and -11 [39]. In a rat sepsis model, Yang et al. demonstrated that Caspase-1 inhibition markedly reduced GSDMD expression in renal tissue, thereby mitigating kidney injury via inhibition of NLRP1 inflammasome activation, reduction of inflammasome-mediated tubular epithelial cell pyroptosis, enhancement of antioxidant enzyme activity, and attenuation of oxidative stress [40]. Concurrently, Ye et al. observed increased expression of soluble death-associated proteins Caspase-11 and GSDMD in renal tubular epithelial cells, which was associated with focal necrotic lesions. Through Caspase-11 knockout experiments, they further confirmed that Caspase-11-mediated focal pyroptosis is critically involved in SA-AKI [41]. Collectively, both pyroptotic pathways contribute significantly to the pathogenesis of SA-AKI.

#### 2.4.3. Autophagy

Autophagy is essential for maintaining intracellular homeostasis during SA-AKI [42]. In CLP-induced sepsis mouse models, markedly increased expression of autophagy-related proteins Beclin1 and LC3-II/LC3-I, accompanied by decreased expression of the autophagy substrate p62, reflects enhanced autophagic activity in SA-AKI [43]. Lipopolysaccharide (LPS) induces autophagy in renal tubular epithelial cells both in vivo and in vitro through TLR4-mediated signaling. To model SA-AKI, researchers administered LPS intraperitoneally to mice with proximal tubule-specific knockout of autophagy-related gene *Atg7* (*Atg7*^KO). Compared to controls, *Atg7*^KO mice exhibited more severe renal dysfunction and parenchymal injury. These findings indicate that autophagy in renal tubular epithelial cells not only prevents endotoxin-induced damage but also regulates downstream signaling of TLR4, thereby mitigating inflammatory responses and tissue injury [44]. Collectively, these studies reveal the protective role of autophagy in SA-AKI and its underlying mechanisms, highlighting its significance in preserving renal tubular epithelial integrity [45] and providing a theoretical basis for developing novel therapeutic strategies. Therapeutic modulation of autophagy may improve renal function in septic patients and reduce the occurrence and progression of acute kidney injury [28].

#### 2.4.4. Ferroptosis

Ferroptosis is a form of cell death driven by iron-dependent lipid peroxidation, with glutathione peroxidase 4 (GPX4) and prostaglandin synthase 2 (PTGS2) widely recognized as canonical markers [46]. Xiao et al. first demonstrated the involvement of ferroptosis in SA-AKI in 2021 [47]. In recent years, studies have increasingly elucidated its role in SA-AKI. The ferroptosis inhibitor biotin A has been proposed as a potential therapeutic or preventive agent for sepsis-related conditions, highlighting ferroptosis as a critical contributor to SA-AKI pathogenesis [48]. Research by Jiarou Li et al. demonstrated that dexmedetomidine reduces GPX4 degradation and effectively inhibits lipid peroxidation and ferroptosis, thereby alleviating SA-AKI symptoms [49]. Additionally, Zhong Xiao et al. reported that NFIL3 is highly expressed in sepsis patients and is closely associated with ACSL4, a central regulator of ferroptosis. Their in vitro and in vivo studies showed that NFIL3 acts as a transcriptional activator of ACSL4. Silencing NFIL3 significantly attenuated reactive oxygen species production, lipid peroxidation, inflammatory responses, and mitochondrial ferroptosis markers [50]. Collectively, these studies indicate that ferroptosis contributes to SA-AKI pathogenesis and suggest that targeting ferroptosis may provide novel therapeutic opportunities. Modulating key molecules such as GPX4, ACSL4, and NFIL3 may limit ferroptosis, thereby offering potential avenues for clinical intervention.

In summary, the pathophysiological mechanisms underlying SA-AKI involve multiple interrelated mechanisms forming complex interactive networks. Microcirculatory dysfunction and inflammatory responses constitute a mutually reinforcing vicious cycle, while alterations in cellular energy metabolism influence the mode of cell death. Evidence indicates that the relative importance of different programmed cell death pathways in SA-AKI is not uniform. Human renal biopsies and various in vivo sepsis models have provided the most direct and compelling evidence for tubular epithelial cell apoptosis and necrotic cell death, identifying these processes as core mechanisms underlying renal dysfunction. In contrast, pyroptosis and ferroptosis are currently documented primarily in animal models and in vitro experiments, and their frequency, temporal characteristics, and pathological contributions in human SA-AKI remain uncertain. These pathways are not mutually exclusive but may dynamically interact, with inflammatory cell death predominating during early tubular injury and apoptotic mechanisms contributing to subsequent functional impairment. This interplay likely explains the heterogeneity observed across studies. Deepening our understanding of these four major pathophysiological mechanisms and unraveling their interrelationships remains a central challenge in current SA-AKI research.

## 3. Drug Therapeutic Targets and Signaling Pathways

**Figure 1:**
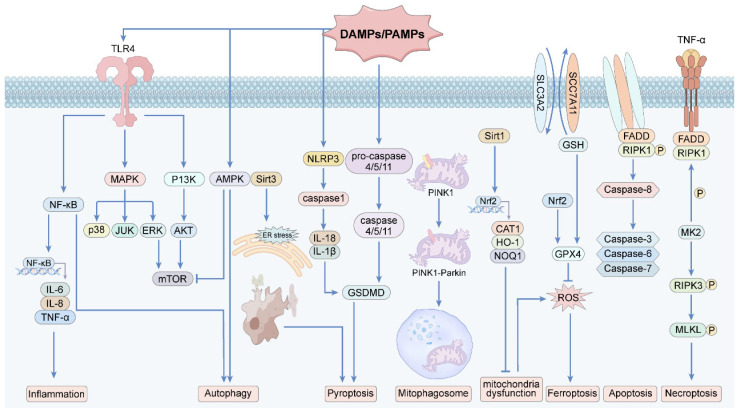
Pathophysiology and signaling pathway mechanism.

This section focuses on the roles of various signaling pathways in the pathophysiology of SA-AKI and reviews current targeted therapies and interventions for these pathways (Table 1). It integrates multiple key signaling pathways in SA-AKI from a systems-network perspective to emphasize their interactions and cross-regulatory relationships (Figure 1).

**Table 1:** Pharmacotherapeutic targets mentioned in relation to SA-AKI.

Therapeutic Agents	Target Pathways	Preclinical Studies	Clinical Trials
Human alkaline phosphatase	Inflammatory response	None	[51]
Ilofotase alfa	Inflammatory response	None	[52]
LMWH	Inflammatory response	None	[53]
CRRT	Inflammatory response	None	[54,55]
Acetaminophen	Anti-pyretic and analgesic	None	[56,57]
Choline	Metabolic disorder	[58]	[58]
RAS inhibition	Hemodynamics	None	[59]
Methylprednisolone	Oxidative stress	[30]	None
N-acetylcysteine	Oxidative stress	[24,25]	None
TMP 195	Class IIa HDAC inhibitor	[60]	None
IL-35	Apoptosis	[61]	None
GYY 4137	Anti-ferroptosis	[62]	None
Biochanin A	Anti-ferroptosis	[48]	None
2,5-Dihydroxyacetophenone	ERK/NF-κB	[63]	None
Byakangelicin	NF-κB	[64]	None
Polygonum cuspidatum Sieb. et Zucc.	NF-κB	[65]	None
TAK-242	TLR 4/NF-κB	[66]	None
Tacrolimus	TLR4/MyD88/NF-κB	[67]	None
SKLB023	TLR4/NFκB/MAPK	[68]	None
Micheliolide	Nrf2/PINK1/Parkin	[69]	None
Rhizoma Coptidis	Nrf2/HO-1	[70]	None
Itaconate	Nrf2	[71]	None
MCTR1	Nrf2	[47]	None
Irisin	SIRT1/Nrf2	[72]	None
Melittin	GPX4/Nrf2	[73]	None
Malvidin	PGC-1α/Nrf2	[74]	None
Recombinant RANKL	OPG/RANKL/RANK/TLR4	[75]	None
TIMP 2	cAMP/NLRP 3	[76]	None
Maresin-1	AMPK/SIRT3	[77]	None
S100A9	Caspase-1/NLRP3	[78]	None
Osajin	IL-33/LPO/8-OHdG/caspase-3	[79]	None
AS and AS- IV	PI3K/AKT	[80]	None
Mesenchymal Stem Cells	Gal-9/Tim-3	[81]	None
Necrostatin-1	LC3-II/p62	[82]	None
HBOT	TGF-β/Smad	[29]	None
ALKBH5	miR-205-5p/DDX 5	[83]	None
Echinocystic acid	PTP 1B	[17]	None
MLR-1023	STAT3	[84]	None

### 3.1. Inflammatory Regulatory Pathways: NF-κB

NF-κB is a crucial transcription factor that regulates the expression of numerous cytokines. NF-κB signaling pathways are activated and play a pivotal role in both in vivo and in vitro models of SA-AK [66,67,85]. In sepsis, NF-κB activation induces the release of proinflammatory cytokines such as TNF-α, IL-1β, and IL-6 [64], riggering downstream inflammatory cascades that subsequently damage renal tubular epithelial cells. Additionally, glycolysis-derived lactate and histone H3K18 lactylation (H3K18la) have been shown to ameliorate renal dysfunction in SA-AKI by attenuating NF-κB-mediated Ezrin K263 lactylation [34], indicating that NF-κB signaling also modulates metabolic processes. Splicosome-associated protein 130 (SAP130), a component of small nuclear ribonucleoproteins, is released from dying cells during apoptosis and programmed necrosis. Peng’s team reported that SAP130 released from ferroptotic tubular epithelial cells promotes M1 macrophage polarization by triggering NF-κB signaling pathways, thereby amplifying ferroptosis in tubular epithelial cells [86]. This demonstrates that NF-κB signaling additionally contributes to cell death processes in SA-AKI. Activation of NF-κB is closely linked to oxidative stress. Increased reactive oxygen species (ROS) induce oxidative stress, activate NF-κB, and subsequently enhance inflammatory responses and cellular injury. Yuan Yang et al. further demonstrated that Polygonum cuspidatum Sieb. et Zucc. extract (PCE) and its active components attenuate SA-AKI by reducing oxidative stress and inhibiting NF-κB signaling, thereby suppressing the production of proinflammatory cytokines in vivo [65]. Based on the available evidence, the NF-κB signaling pathway functions as a “central inflammatory integration hub” in SA-AKI rather than serving as a solitary initiating mechanism. Its activation shows remarkable consistency across multiple SA-AKI animal models and human samples, placing it at the nexus of inflammation, oxidative stress, and cell death pathways. Consequently, NF-κB serves as a critical node for multi-targeted intervention strategies rather than a solitary therapeutic target.

### 3.2. Oxidative Stress and Cytoprotective Pathways: Nrf2

Nuclear Factor Erythroid 2-Related Factor 2 (Nrf2) is a crucial transcription factor that activates the antioxidant response element, thereby inducing the expression of various antioxidant enzymes [87]. In SA-AKI, Nrf2 activation significantly alleviates oxidative stress and protects renal tubular epithelial cells from ROS-induced injury. Berberine extract has been reported to enhance Nrf2 nuclear translocation, upregulate HO-1 protein and PPARα mRNA expression, reduce NOS2 activity, and improve renal function and histopathological injury in SA-AKI [70]. The Nrf2/HO-1 signaling pathway suppresses iron-dependent cellular injury in proximal tubular epithelial cells [88]. Ji Xiao et al. first identified ferroptosis, an iron-dependent form of cell death, as being mediated by Nrf2 in SA-AKI [47]. Concurrently, irisin has been reported to alleviate ferroptosis via activation of the SIRT1/Nrf2 pathway, thereby protecting against SA-AKI [72]. Furthermore, Nrf2 competitively binds to NF-κB DNA-binding sites, inhibiting NF-κB nuclear translocation and suppressing the expression of proinflammatory cytokines, including TNF-α, IL-1β, and IL-6, which contributes to attenuating inflammatory responses and protecting renal function [89]. Nrf2 signaling also plays a central role in mitigating oxidative stress in SA-AKI. Studies on itaconic acid 4-octyl ester (OI) have demonstrated that OI activates Nrf2, reducing oxidative stress, attenuating renal tubular cell apoptosis, and ameliorating mitochondrial dysfunction [71,90]. Similarly, activation of the Nrf2/HO-1 pathway improves renal function and mitigates histopathological injury in septic mice by alleviating oxidative stress [91]. Unlike pro-inflammatory pathways, the Nrf2 signaling pathway serves predominantly as an intrinsic protective and adaptive response in SA-AKI. Extensive experimental studies indicate that Nrf2 activation can mitigate oxidative stress, inhibit ferroptosis, and improve mitochondrial function, although such activation generally occurs subsequent to the onset of renal injury. Direct evidence is currently lacking to establish Nrf2 as a pathogenic driver of SA-AKI, and it likely sets the kidney’s threshold for tolerating inflammatory and metabolic stress. Consequently, the Nrf2 pathway acts primarily as a disease-modifying mechanism in SA-AKI rather than as a primary pathogenic pathway [92,93].

### 3.3. Cell Death and Stress Response Pathways: MAPK

The MAPK signaling pathway comprises three major branches: ERK, JNK, and p38 MAPK. Activation of these pathways primarily regulates the expression of proinflammatory cytokines, such as TNF-α, IL-1β, and IL-6, exacerbating systemic and local inflammatory responses and causing injury to renal tubular epithelial cells. Among these, activation of the JNK and p38 MAPK pathways is closely associated with apoptosis and necrosis [94], further aggravating renal injury. MAPK signaling activation is also linked to oxidative stress. Increased ROS can activate the JNK and p38 MAPK pathways, amplifying oxidative stress and inducing cellular injury. Hui Li et al. demonstrated that the synthetic small-molecule compound SKLB023 improves survival in SA-AKI mice and attenuates renal histopathological injury, inflammation, and apoptosis in both CLP- and LPS-induced SA-AKI models, primarily through MAPK pathway activation [68]. In summary, the MAPK signaling pathway in SA-AKI predominantly functions as a downstream effector of inflammatory and oxidative stress signals, mediating cell fate decisions rather than independently initiating injury. Its activation is closely associated with tubular epithelial cell apoptosis and necrosis, yet MAPK activation often depends on upstream TLR/NF-κB signaling and ROS accumulation. This suggests that MAPK serves primarily in the execution phase of cell death rather than determining disease initiation. Current MAPK-targeted interventions remain largely confined to animal models, and their specificity and safety in human SA-AKI require further validation.

### 3.4. Metabolism-Related Pathways: PI3K/AKT

The PI3K/AKT signaling pathway is a canonical intracellular signaling cascade that transduces extracellular stimuli via sequential protein phosphorylation events, thereby regulating cellular behavior and function. Research findings indicate that Astragalus and its active component astragaloside IV effectively attenuate tubular injury in murine sepsis models, including reducing vacuolation of tubular epithelial cells, preventing loss of the brush border, and ameliorating mitochondrial ultrastructural alterations. Additionally, Astragalus and astragaloside IV suppress the elevated expression of kidney injury molecule-1 (KIM-1). In vitro experiments demonstrated that astragaloside IV effectively protects human proximal tubular epithelial cells, counteracting LPS-induced reductions in cell viability. Western blotting and immunohistochemical analyses further confirmed that both Astragalus and astragaloside IV activate the PI3K/AKT signaling pathway in both in vivo and in vitro settings, thereby promoting cell survival and reducing apoptosis [80]. Collectively, the PI3K/AKT pathway primarily contributes to tubular epithelial cell survival and stress adaptation in SA-AKI rather than mediating non-specific injury mechanisms. Its activation is observable across multiple AKI subtypes, likely representing a generalized protective response of tubular epithelial cells to inflammatory and metabolic stress. Although it demonstrates clear nonprotective effects in animal models, its lack of disease specificity may limit its translational potential as an independent therapeutic target. Therefore, PI3K/AKT is more appropriately regarded as a supportive or auxiliary regulatory pathway rather than a core pathogenic mechanism in SA-AKI [95].

### 3.5. Autophagy and Mitochondrial Quality Control Pathways: PINK1-Parkin

PINK1 and Parkin are central regulators of mitophagy. Upon mitochondrial damage, PINK1 accumulates on the outer mitochondrial membrane, recruiting Parkin and thereby activating Parkin’s E3 ubiquitin ligase activity. Parkin ubiquitinates outer mitochondrial membrane proteins, facilitating their recognition and removal by autophagosomes and thereby eliminating dysfunctional mitochondria [96]. Activation of the PINK1/Parkin signaling pathway also mitigates mitochondria-dependent apoptosis and necrosis through enhanced mitophagy. Lei et al. demonstrated that targeting the PINK1/Parkin axis to enhance mitophagy alleviates SA-AKI [69]. Peng et al. further revealed that macrophage migration inhibitory factor (MIF) directly binds to PINK1, disrupting its interaction with Parkin. This inhibition of mitophagic initiation leads to extensive tubular epithelial cell apoptosis. Their findings not only confirm the role of PINK1/Parkin in SA-AKI but also suggest the therapeutic potential of MIF inhibitors or novel mitophagy activators in this condition [97]. In summary, PINK1–Parkin-mediated mitophagy is increasingly recognized as a pivotal hub linking energy metabolism, oxidative stress, and cell death in SA-AKI. Unlike simple anti-inflammatory strategies, this pathway directly targets energy homeostasis and mitochondrial integrity in tubular cells, demonstrating consistent protective effects across multiple experimental models. However, its temporal dynamics and optimal intervention window in human SA-AKI remain unclear, limiting current clinical translation. These observations suggest that the PINK1–Parkin pathway may represent a novel therapeutic direction with potential disease-modifying capabilities.

### 3.6. Energy Metabolism and Autophagy Pathways: AMPK

AMPK is a highly conserved master regulator of metabolism, which is activated under conditions of energy stress, restoring cellular and systemic energy homeostasis [98]. AMPK activation promotes SIRT3 expression and activity. SIRT3 is a mitochondria-localized deacetylase that activates multiple antioxidant enzymes through deacetylation and modulates mitochondrial protein activity to preserve normal mitochondrial function. Activation of the AMPK/SIRT3 pathway protects mitochondrial integrity and mitigates apoptosis, making it a potential therapeutic target for AKI [99]. In SA-AKI, activation of the AMPK/SIRT3 pathway also significantly alleviates oxidative stress, thereby shielding renal tubular epithelial cells from ROS-induced injury [100]. Research has further revealed that Maresin-1, a lipid mediator promoting inflammation resolution, attenuates SA-AKI via activation of the AMPK/SIRT3 signaling pathway [77]. In summary, the AMPK signaling pathway in SA-AKI primarily represents an adaptive cellular response to energy stress rather than an inflammation- or infection-specific pathway. Its activation helps maintain mitochondrial function and limits excessive cell death, although it is often contingent on upstream metabolic stress and microcirculatory dysfunction. Consequently, AMPK is more likely to modulate tubular cell resilience in the context of sepsis rather than drive disease onset. This characteristic positions it as a potential prognostic regulator and a target for combination therapeutic strategies.

### 3.7. ncRNA-Related Signaling Pathways

In recent years, research on non-coding RNA (ncRNA)-related signaling pathways has elucidated the roles of various ncRNAs in the pathophysiology of SA-AKI. NcRNAs regulate gene expression by inhibiting target mRNA translation or promoting its degradation, thereby modulating inflammation, programmed cell death, and oxidative stress in SA-AKI [101]. Although ncRNA-related pathways provide new insights into the complex regulatory networks of SA-AKI, most evidence currently derives from in vitro experiments and rodent models. Inconsistencies exist among studies regarding the types of ncRNAs reported and their functional orientations, and their stability and functional relevance in human SA-AKI remain to be validated. Therefore, ncRNAs are currently more suitable as candidates for mechanistic studies and potential biomarkers rather than as established therapeutic targets. Table 2 summarizes the major ncRNAs involved in SA-AKI. For clarity, ncRNAs are grouped by functional role: protective ncRNAs are listed first, followed by deleterious ncRNAs.

**Table 2:** Signaling pathways associated with ncRNA in SA-AKI.

ncRNA	Target Pathways	Pathophysiological Mechanism	Effect	References
Protective				
lncRNA NORAD	miR-155-5p/PDK1	Repair of glucose metabolism inhibition	Relieve SA-AKI	[102]
circ_001653	KEAP1/Nrf2/HO-1	Reduces apoptosis, inflammation and oxidative stress	Effective attenuation of SA-AKI	[103]
siRNA nanoparticles	NF-κB, p65	Inflammatory	Protection of SA-AKI	[104]
hsa_Circ_0072463 mmu_Circ_26986	miRNA-29b-1-5p/PAK7	Inhibition of apoptosis	Mediating SA-AKI	[105]
Deleterious				
miR-124-3p.1	miR-124-3p.1/LPCAT3	Affects lipid metabolism, leading to ferroptosis	Mediating SA-AKI	[106]
Circ_0006944	miR-205-5p/UBL4A	Exacerbates apoptosis, inflammation and oxidative stress	Exacerbation of SA-AKI	[107]
circ-BNIP3L	miR-370-3p/MYD88	Cell growth inhibition, inflammation and oxidative stress	Exacerbation of SA-AKI	[108]
miR-205-5p	miR-205-5p/DDX5	Promotion of EMT	Facilitating the AKI-CKD transition	[83]
miRNA-223-3p	microRNA-223-3p/SGK1	Promotes apoptosis and inflammatory responses	Exacerbation of SA-AKI	[31]
LncRNA MIAT	PTBP 1/BECN 1	Activation of cellular autophagy, apoptosis and inflammation	Exacerbation of SA-AKI	[109]
circ_0114428	miR-215-5p/TRAF6/NF-κB	Cell proliferation, apoptosis and inflammatory damage	Highly expressed in SA-AKI patients and LPS-treated HK-2 cells	[110]
circ_0114428	miR-370-3p/TIMP 2	Cellular inflammation and apoptosis	Promoting SA-AKI progress	[111]
circ_0001714	miR-129-5p/TRAF 6	Apoptosis and inflammation	Exacerbation of LPS-induced renal tubular epithelial cell injury	[112]

Overall, the signaling pathways described in Part II function at distinct hierarchical levels in SA-AKI. Inflammatory pathways such as NF-κB constitute upstream integration hubs, whereas pathways like MAPK and PI3K/AKT primarily mediate cellular responses. Nrf2, AMPK, and PINK1–Parkin mediate cellular adaptation and recovery following injury. Understanding the hierarchical relationships among these pathways is crucial for clarifying the mechanisms underlying previous clinical trial shortcomings and for designing future combination-targeted strategies. This review systematically summarizes the major signaling pathways associated with SA-AKI. Due to differences in the roles of various pathways in pathogenesis and the depth of research, the extent of discussion devoted to each pathway varies accordingly. For example, pathways related to inflammation and oxidative stress play central roles in SA-AKI and are supported by extensive clinical and experimental evidence, warranting more detailed discussion. Concurrently, other signaling pathways also participate in SA-AKI regulation, though their mechanisms are less complex or less extensively characterized in the literature.

## 4. Biomarker

Biomarkers play a crucial role in the diagnosis, treatment, and prognostic evaluation of SA-AKI. Although multiple emerging biomarkers show promise for the early detection of SA-AKI, their clinical application remains limited by challenges such as insufficient specificity, marked interpatient variability, and lack of standardized detection protocols. Consequently, these markers (Table 3) are currently best utilized as tools for risk stratification and mechanistic research, rather than as standalone diagnostic criteria.

### 4.1. Glomerular Filtration Markers

Creatinine, a traditional indicator for evaluating renal function, is primarily used to measure glomerular filtration rate. However, changes in serum creatinine levels often lag behind actual renal impairment and are influenced by factors such as muscle mass, age, and gender, leading to limited sensitivity and specificity. Blood urea nitrogen (BUN) is another indicator reflecting GFR, but its levels are also affected by protein intake and liver function, thereby limiting its specificity. In contrast, cystatin C, a low-molecular-weight protein continuously produced by all nucleated cells and unaffected by muscle mass, is considered a more sensitive marker for evaluating glomerular filtration. It provides an earlier and more reliable reflection of renal function than serum creatinine. When combined with other biomarkers, cystatin C offers substantial prognostic utility for predicting readmission rates and both short-term and long-term mortality. Urinary albumin serves as a crucial indicator for evaluating damage to the glomerular filtration barrier, with high sensitivity for the early detection of kidney injury and effective assessment of the extent of glomerular impairment. Proenkephalin (PENKID), an endogenous opioid protein, has been extensively studied as a potential renal function biomarker in ICU settings. As a marker of glomerular filtration, changes in PENKID may precede alterations in serum creatinine or other GFR-related markers, enabling earlier detection of changes in renal function [20,113].

In summary, while creatinine and BUN remain commonly used tools for renal function assessment, novel biomarkers such as cystatin C, urinary albumin, and proenkephalin offer significant advantages by enabling earlier and more precise renal monitoring. These markers not only enhance the sensitivity of detecting renal function changes but also offer complementary information to guide clinical decision-making and patient management.

### 4.2. Markers of Tubular Injury

Neutrophil Gelatinase-Associated Lipocalin (NGAL) is a 25 kDa protein primarily localized in neutrophil granules and specific tissues, including renal tubular epithelium and cardiomyocytes. NGAL undergoes glomerular filtration and is reabsorbed in the proximal tubule, serving as an early marker of tubular injury and renal function decline. It can detect renal injury prior to changes in traditional renal function indicators and correlates with residual renal function in dialysis patients. Kidney Injury Molecule-1 (KIM-1) is a transmembrane glycoprotein expressed by proximal tubule cells following hypoxic injury, demonstrating high specificity for ischemic acute kidney injury. KIM-1 exhibits both high sensitivity and specificity for early detection and severity assessment of renal injury but lacks specificity for prerenal azotemia, chronic kidney disease, or contrast-induced nephropathy. Liver-type fatty acid-binding protein (L-FABP) facilitates the transport of free fatty acids to mitochondria and peroxisomes, mitigating damage from reactive oxygen species. Its expression is upregulated during ischemia–reperfusion injury, potentially reflecting sepsis severity and serving as a monitor for polymyxin hemoperfusion therapy efficacy. N-acetyl-β-D-glucosaminidase (NAG), a lysosomal enzyme present in proximal tubule cells, shows elevated urinary levels after tubular injury, reflecting the extent of renal tubular damage. Urinary retinol-binding protein (RBP), a low-molecular-weight protein synthesized in the liver, undergoes glomerular filtration and tubular reabsorption; alterations in urinary RBP facilitate early detection of tubular injury. Similarly, β2-microglobulin, another low-molecular-weight protein reabsorbed in renal tubules, exhibits elevated urinary levels in the presence of tubular damage. Collectively, these biomarkers demonstrate high early sensitivity and provide valuable information for assessing renal tubular injury [20,113].

### 4.3. Cell Cycle Arrest Markers

Tissue Inhibitor of Metalloproteinases-2 (TIMP-2) and Insulin-like Growth Factor Binding Protein-7 (IGFBP7) are involved in G1 phase cell cycle arrest during the early stages of cellular injury. The combined measurement of TIMP-2 and IGFBP7 has demonstrated significant advantages in predicting acute kidney injury (AKI) risk in critically ill patients and those undergoing cardiac surgery. This biomarker combination is particularly effective in assessing the prognosis of sepsis-associated AKI (SA-AKI), enhancing early risk stratification and enabling timely clinical interventions. Consequently, the use of TIMP-2·IGFBP7 may improve patient outcomes and survival rates by facilitating proactive management strategies [114,115].

### 4.4. Endothelial Injury Markers

Angiopoietin-1 (Ang-1) stabilizes endothelial cells and exerts vascular-protective effects, whereas angiopoietin-2 (Ang-2) promotes vascular leakage and may exacerbate sepsis. The balance between Ang-1 and Ang-2 is essential for maintaining vascular homeostasis. Vascular endothelial cadherin (VE-cadherin), a critical component of intercellular junctions, is closely associated with severe acute kidney injury requiring renal replacement therapy. Disruption of VE-cadherin compromises endothelial barrier function, impairing normal renal filtration and reabsorption. Soluble thrombomodulin (sTM), a thrombin receptor expressed on the endothelial surface, is released into the circulation upon endothelial activation or injury. Elevated plasma sTM levels in critically ill septic patients serve as an independent predictor of sepsis-associated acute kidney injury, facilitating early identification of high-risk individuals and guiding timely clinical interventions. Collectively, Ang-1/Ang-2, VE-cadherin, and sTM represent key biomarkers for evaluating endothelial integrity, renal pathophysiology, and prognosis in sepsis, providing valuable information for clinical decision-making [116].

### 4.5. Inflammatory Markers

Soluble triggering receptor expressed on myeloid cells-1 (sTREM-1) is an activation receptor selectively expressed on neutrophils and monocytes, closely linked to the inflammatory response triggered by bacterial infections. It plays a central role in the host immune defense, and elevated sTREM-1 levels typically reflect active infection or systemic inflammation [117]. Interleukin-6 (IL-6) is a multifunctional pro-inflammatory cytokine involved in acute-phase responses, immune regulation, and inflammatory processes. Beyond its role in host defense, IL-6 contributes to the development and persistence of inflammation under pathological conditions. Interleukin-18 (IL-18), another key pro-inflammatory cytokine, is particularly detectable in urine following acute ischemic injury of proximal tubular cells. Released by injured tubular epithelial cells, IL-18 exhibits high early sensitivity for assessing renal damage and associated inflammatory responses [77]. Collectively, sTREM-1, IL-6, and IL-18 serve as valuable biomarkers for monitoring kidney injury and its inflammatory component. These markers not only facilitate early detection of renal dysfunction but also provide critical insights into disease progression and the design of timely, targeted therapeutic strategies, particularly in acute kidney injury accompanied by systemic inflammation.

### 4.6. Hemodynamic-Related Markers

Renin is an enzyme secreted by the kidneys in response to reduced tissue perfusion, sympathetic activation, or hypoxic stress. Beyond serving as a marker of tissue perfusion, renin has been shown to outperform lactate in predicting mortality among critically ill patients. As a key biomarker in sepsis-associated acute kidney injury (SA-AKI), renin provides valuable guidance for vasopressor therapy in patients with shock. Clinical and experimental studies indicate that administration of angiotensin II lowers renin concentrations, improves renal hemodynamics, and enhances AT1 receptor (AT1R) signaling, thereby directly influencing the pathophysiology of SA-AKI [118]. Dipeptidyl peptidase 3 (DPP3) is a cytoplasmic, zinc-dependent metallopeptidase widely expressed across human tissues and implicated in the regulation of immune responses and oxidative stress. DPP3 can cleave multiple peptides, including angiotensin, enkephalins, and endorphins, and has been identified as a key regulator within the renin-angiotensin system (RAS), targeting Ang II, Ang-(1–7), and Ang-(1–5) [119]. These findings suggest that modulating DPP3 activity may represent a novel therapeutic approach for improving outcomes in SA-AKI [118]. In summary, renin functions both as a critical indicator of tissue perfusion and a central mediator of SA-AKI pathophysiology. Monitoring renin levels and understanding their modulation by angiotensin II provides actionable insights for clinical management. Concurrently, further investigation into DPP3’s regulatory role within the RAS may reveal innovative therapeutic strategies to mitigate renal injury in sepsis.

**Table 3:** Biomarkers of SA-AKI.

Type	Name	Feature	Strengths/Weaknesses
Glomerular filtration markers	Cr	Measuring glomerular filtration rate	Quick and convenient; Sensitivity and specificity are relatively limited due to various factors such as muscle mass, age, and gender.
	BUN	Same as above	Quick and convenient; Affected by factors such as protein intake and liver function status, resulting in poor specificity.
	Cystatin C	Low molecular weight proteins	Early sensitivity and timeliness; When used in combination with other biomarkers, it provides significant additional prognostic value for predicting readmission rates as well as short- and long-term mortality.
	u-Alb	Assessing damage to the glomerular filtration barrier	Early Sensitivity; Capable of effectively assessing the degree of glomerular damage.
	PENK	Endogenous opioid proteins	Appears earlier than serum creatinine or other GFR-related markers; As a potential biomarker in the intensive care unit setting
Renal tubular injury markers	NGAL	Primarily found in specific organ sites such as neutrophil granules and renal tubular epithelial cells.	Early Markers of Tubular Injury and Renal Function Deterioration; Detectable prior to changes in renal function indicators, these markers correlate with residual renal function status in dialysis patients.
	KIM-1	A transmembrane glycoprotein expressed by proximal tubule cells following hypoxic injury	Highly specific for ischemic acute kidney injury; Highly sensitive and specific in the early stages, making it very useful for assessing the severity of kidney injury; Not specific for prerenal azotemia, chronic kidney disease, or contrast-induced nephropathy [120].
	L-FABP	Transport free fatty acids to mitochondria and peroxisomes for metabolism	Reflects the severity of sepsis; Monitors the efficacy of polymyxin hemoperfusion therapy.
	NAG	A lysosomal enzyme present in proximal tubule cells	Reflects the degree of renal tubular injury.
	RBP	Low molecular weight proteins	Primarily synthesized in the liver, filtered through the glomerulus, and reabsorbed in the renal tubules; Used for early assessment of tubular injury.
	β2-MG	Low molecular weight proteins	Primarily reabsorbed in the renal tubules; Elevated urinary levels indicate the presence of renal tubular damage.
cell cycle block marker	[TIMP-2] * [IGFBP7]	Participates in the G1 phase cell cycle arrest process during the early stages of cellular damage	Demonstrated significant advantages in predicting acute kidney injury risk in critically ill patients and those undergoing cardiac surgery; Effectively assessed the prognosis of SA-AKI patients [114]; Enhanced the accuracy of SA-AKI risk prediction.
Markers of endothelial damage	Ang-1	Stabilize endothelial cells to exert a protective effect	It plays a crucial role in regulating vascular stability, and its equilibrium is essential for maintaining normal vascular function.
	Ang-2	Promotes vascular leakage, which may exacerbate sepsis	Same as above.
	VE-cadherin	An essential component of endothelial cell–cell junctions	Severe acute kidney injury requiring renal replacement therapy is closely associated with potential impairment of endothelial barrier function, which may subsequently affect the kidney’s normal filtration and reabsorption capabilities.
	sTM	The receptor for thrombin is typically expressed on the surface of endothelial cells	Identify high-risk patients early and guide the development of clinical intervention measures.
inflammation marker	sTREM-1	An activation receptor selectively expressed on the surface of neutrophils and monocytes	Elevated levels typically indicate the presence of an active infection or inflammatory process.
	IL-6	A multifunctional pro-inflammatory cytokine	Participates in the body’s defense mechanisms against infection and promotes the development and persistence of inflammation under various pathological conditions.
	IL-18	An important pro-inflammatory cytokine	It can be detected in urine, particularly following acute ischemic proximal tubular injury. Released by renal tubular epithelial cells after kidney injury, it exhibits high early sensitivity and can be used to assess the severity of renal injury and the accompanying inflammatory response.
Haemodynamically relevant markers	REN	An enzyme secreted by the kidneys in response to reduced tissue perfusion, sympathetic nervous system activation, or hypoxic metabolism	It serves as a marker of tissue perfusion and outperforms lactate in predicting mortality among ICU patients, enabling guidance for vasopressor therapy in shock patients.
	DDP3	A cytoplasmic, zinc-dependent metalloprotein widely present in human cells	It is closely associated with the regulation of immune responses and oxidative stress. Possessing the ability to cleave various peptides, including angiotensin, enkephalins, and endorphins, it forms part of the RAS regulatory network.
Other types	AFM	A key gene significantly associated with monocyte infiltration	Negatively correlated with the recruitment of monocytes and the release of various inflammatory factors in AKI kidneys; Closely associated with the occurrence and progression of SA-AKI [121].
	STAT3 and IFITM3	Genes involved in coagulation and inflammation	In vitro studies indicate that STAT3 and IFITM3 partially regulate endothelial coagulation and inflammatory activation; A novel endothelial therapeutic target identified for sepsis [122].
	Obesity	is a known risk factor for chronic kidney disease	Obesity is associated with a higher risk of early SA-AKI. The presence of SA-AKI modifies the association between obesity and clinical outcomes [123].
	Cell-free DNA	Free DNA fragments circulating in the extracellular space	A predictive model for mortality, SA-AKI, and RRT risk in sepsis patients has the potential to significantly improve outcomes, offering distinct advantages over traditional diagnostic approaches [124].
	Plasma SCUBE2	A secreted calcium-binding protein that plays a role in various physiological processes, including intercellular communication, angiogenesis, and development	Lower SCUBE 2 plasma levels correlate with elevated proinflammatory factors and impact survival outcomes; It may serve as a potential biomarker for predicting prognosis in patients with SA-AKI [125].
	TyG	It is a comprehensive indicator for assessing insulin resistance	Closely associated with SA-AKI and length of hospital stay in patients with sepsis; A potential predictor of SA-AKI and length of hospital stay in sepsis cases [126].
	Plasma oxidative lipidomics	It is an important bioactive lipid mediator produced during infection that regulates oxidative stress and inflammatory responses	5(S),12(S)-DiHETE, 5-isoPGF2VI, 5,6-DiHETrE, 11,12-EET, and 9,10-DiHOME exhibit high sensitivity and specificity [127].
	LAR	It is an indicator that assesses certain disease states by calculating the ratio of lactate dehydrogenase (LDH) activity to serum albumin (ALB) concentration in the blood	LAR is associated with poor prognosis in SA-AKI patients. Higher LAR correlates with increased 28-day, 90-day, and in-hospital mortality rates [128].
	SII	It is an indicator that comprehensively reflects the body’s systemic inflammatory state and immune status	Both low SII and high SII are associated with an increased risk of short-term mortality in SA-AKI [129].
	SIRI	It is an indicator used to assess the systemic inflammatory state of the body	Can serve as a comprehensive biomarker for predicting all-cause mortality in patients with severe SA-AKI.
	APTT	A screening test used to detect the activity of coagulation factors in the intrinsic coagulation pathway	Elevated baseline APTT is associated with increased risk of 28-day mortality suggesting poor prognosis in SA-AKI [130].
	Galectin-3	It is a multifunctional protein belonging to the galectin family	Become a key pathogenic factor and potential therapeutic target for SA-AKI [131].

Note: [TIMP-2] * [IGFBP7] indicates the product of TIMP-2 and IGFBP7 concentrations.

## 5. Current Challenges and Limitations

Despite substantial advances in elucidating the mechanisms underlying SA-AKI, significant challenges remain. As summarized in Table 1, most potential therapeutic strategies are still at the preclinical stage and have not progressed to randomized controlled clinical trials. Key aspects of the pathogenesis remain incompletely understood, and available therapeutic options are relatively limited. Moreover, the safety, efficacy, and translational potential of many experimental interventions require further rigorous clinical validation before routine application.

Animal models often fail to fully recapitulate the complex pathophysiology of human SA-AKI due to species-specific differences in immune responses, metabolic regulation, and organ susceptibility. Most preclinical studies rely on young, healthy rodents and simplified sepsis models, such as cecal ligation and puncture (CLP) or endotoxemia, which do not reflect critical aspects of human sepsis, including advanced age, chronic comorbidities, polypharmacy, and prolonged disease course. Consequently, therapeutic effects observed in these experimental settings often overestimate clinical efficacy. Timing of intervention also poses a major translational challenge: in animal studies, treatments are usually administered before or immediately after sepsis induction, whereas patients in clinical practice often present after severe organ dysfunction has already developed. This temporal mismatch may partly explain the limited success of therapies targeting early inflammatory pathways in human trials. Additionally, extensive cross-talk between signaling pathways—for example, the competitive interplay between NF-κB and Nrf2—further complicates targeted interventions, as inhibition of a single node may inadvertently disrupt compensatory or adaptive mechanisms.

Clinically, patient heterogeneity significantly limits the efficacy of monotherapies in SA-AKI. SA-AKI is not a single disease entity but rather a highly heterogeneous syndrome. Considerable variations exist among patients in terms of infection sources, host immune status, baseline renal function, and genetic background. Such differences may result in the same molecular pathways exerting inconsistent pathological effects across individuals. However, most preclinical studies fail to model or stratify these variations, which restricts the translational applicability of their findings.

In recent years, advances in big data analytics and multi-omics technologies have provided cutting-edge tools for addressing these challenges in SA-AKI research. Machine learning–based risk prediction models can integrate clinical indicators—such as blood pressure, serum creatinine, and inflammatory markers—with laboratory data to identify high-risk patients early, enabling precise stratification and individualized intervention [132,133].

Concurrently, multi-omics strategies, which integrate transcriptomic, proteomic, metabolomic, and other datasets, allow comprehensive mapping of the molecular networks underlying SA-AKI, thereby identifying key regulatory pathways and potential therapeutic targets. For example, combined metabolomics–proteomics analyses have revealed early metabolic alterations in SA-AKI, highlighting potential avenues for targeted intervention [134]. These approaches not only deepen mechanistic understanding but also provide innovative strategies for early diagnosis, risk prediction, and personalized treatment [135].

In summary, the limited success in translating SA-AKI research into clinical practice arises from multiple converging factors, including simplified animal models, misaligned timing of interventions, and patient heterogeneity. Future research should emphasize precise disease classification, dynamic monitoring, and multi-target combination therapies to improve the translational success of preclinical findings and ultimately enhance clinical outcomes.

## 6. Conclusions

Sepsis-associated acute kidney injury, as a complex and life-threatening clinical syndrome, highlights the need for the development of novel therapeutics targeting new pathways, as well as the integration of multi-omics approaches and artificial intelligence, which represent promising directions for future treatment strategies.

## Data Availability

No new data were created or analyzed in this study. Data sharing is not applicable to this article.
